# Teaching an Adult with ASD Independent Toileting Skills

**DOI:** 10.3390/healthcare12232374

**Published:** 2024-11-26

**Authors:** Angela Deoki, Vicki Madaus Knapp

**Affiliations:** 1 Department of Behavioral Science, Daemen University, 4380 Main St., Amherst, NY 14226, USA; 2 The Summit Center, 150 Stahl Rd., Getzville, NY 14068, USA

**Keywords:** toileting skills, toilet training, autism spectrum disorder, adult

## Abstract

**Background:** Independence with the toilet is an important life skill. Individuals with Autism Spectrum Disorder (ASD) may present with several deficits that impair their ability to independently use the toilet and often individuals with ASD require more support than is provided in typical toilet training methods, including behavioral toilet training methods. This current study is a demonstration of the use of an intensive toilet training procedure with one eighteen-year-old adult male with ASD and below-average levels of adaptive functioning to successfully void in the toilet and reduce accidents. **Methods**: A package of behavioral toilet training procedures was implemented for 11 weeks in a school setting. This package included intensive toilet training, a urine alarm, a toileting schedule, verbal praise, edible reinforcement, and dry pants checks. School staff were trained to implement this procedure with behavioral skills training (BST). **Results**: Intensive toilet training was effective in eliminating the number of accidents while increasing the number of successes, thus increasing his independence with toileting. **Conclusions**: Increasing independence with toileting is an important life skill and can increase the likelihood that an individual would be accepted by an adult services placement.

## 1. Introduction

The ability to use the toilet independently is an essential life skill [1,2,3]. An individual who urinates in places other than the toilet during the daytime and nighttime is considered to present with functional incontinence (FI) concerning the voiding of urine [4]. A person who displays FI does not hold urine and wait to void in a toilet. Further, urinary incontinence refers to the continuous or intermittent leaking of urine and can include daytime urinary incontinence (DUI) and nocturnal enuresis (NE) [4,5]. The prevalence of NE in people with an autism spectrum disorder (ASD) ranges from 2% to 41% and up to 90% of patients with ASD who see a urologist present with NE [5]. The rates of DUI in people with ASD have been extrapolated from samples that have been highly selected and range from 4.3% to over 25% and up to 55% of patients with ASD who see a neurologist [5]. Other studies suggest that the rate of DUI in people with Intellectual and Developmental Disabilities (IDDs) varies and ranges between 23% and 86% [6]. Additionally, an individual who passes feces in places other than the toilet during the day or night repeatedly is considered to present with functional encopresis (FE) [7].

Typical toilet training methods are led by parents and caregivers and include demonstrating or modeling the toileting skills for the child, allowing the child to sit on a small “potty chair”, and allowing the child to learn at their own pace [8,9]. Some traditional toilet-training methods encourage the use of rewards for the appropriate use of the toilet [10,11]. Conventional toilet training might work for people with ASD, but they often require more support and more intensive and repeated trials for learning. One key feature of the diagnosis of ASD is restricted and repetitive behavior, which could take the form of insistence on sameness and could make toilet training difficult. Individuals with ASD often benefit from specialized interventions and structured learning situations where tasks are broken down into smaller pieces with many repeated opportunities to practice a skill [12,13,14]. There are many effective behavioral methods that can be used to help an individual with ASD achieve independence with using the toilet, which include the use of antecedent, consequence, and prompting techniques [2]. In a critical review of the toilet training literature for individuals with ASD and other developmental disabilities, Kroeger and Burnworth (2009) found that the techniques of graduated guidance, reinforcement-based training, scheduled sittings, elimination schedules, punishment procedures, hydration, the manipulation of stimulus control, nighttime training, and parenting and video modeling were the most effective and prevalent in teaching toilet training [2]. Simon and colleagues (2022) found that an understanding and consideration of the learner’s developmental level may facilitate the toilet training process for learners with ASD [3]. Further, specific considerations have been outlined for teaching learners with ASD to use the toilet independently in a school setting [15].

There is much research on the topic of toilet training or the reduction in or elimination of enuresis and/or encopresis in children with intellectual and developmental disabilities (IDDs), including ASD [1,3,7,16,17,18]. Notably, Azrin and Foxx (1971) developed a comprehensive toilet training procedure referred to as Rapid Toilet Training (RTT), which could be completed in a home or school setting [16]. In this study, nine incontinent adults who were residents in institutional care for their physical and developmental disabilities were taught to use the toilet. The toilet training procedures included the use of an enuresis alarm (explained in more detail below), increased fluid intake, scheduled visits to the toilet, reinforcement for staying dry/clean, reinforcement for elimination in the toilet, and an overcorrection procedure for accidents [16]. More recent applications of RTT do not include overcorrection [1,18].

A common behavioral method that can be used in conjunction with intensive toilet training or by itself, utilized by Azrin and Foxx (1971), is an enuresis alarm or urine alarm [13,16]. A urine alarm should be used with other behavioral methods, such as positive reinforcement and modeling. A urine alarm is a device that can carry a wire, wireless, or a pad-type alarm. Both the wired and wireless devices are small and can easily clip on to a diaper, pull up, or underwear. With the pad-type alarm or the bell and pad method, the sensor is on a pad, which is placed under the child for them to sleep on; the pad then detects the moisture and an alarm sounds [13,19]. The purpose of the alarm is to aid in the process of toilet training by detecting moisture at the first instance, which teaches the wearer to respond once they hear the sound; an auditory response is provided as the alarm sounds, signaling moisture is occurring and potentially allowing for the void to be continued in the toilet [13,20].

Individuals who do not use the toilet independently may require assistance, which could lead to financial hardship and the risk of abuse [21]. Individuals with disabilities who do not use the toilet independently may be dependent on diapers, which can be costly. A common prerequisite for participating in adult services programs and other social groups is the ability to use the toilet independently. If an individual has not learned to use the toilet independently by the time they transition from school to adult services, they may not be accepted to a placement and the caregivers may have to pay for additional care out-of-pocket. Using the toilet independently allows for staff to not have to be directly in the bathroom with an individual and therefore protects and sustains their autonomy. In general, individuals with disabilities, such as ASD, are more at risk of abuse; therefore, it may follow that if one needs support in the bathroom when they are already in a vulnerable position, they are more susceptible to abuse [21].

Research in teaching adults with ASD to use the toilet independently is limited and focused on young children, and studies include several limitations, including the use of small sample sizes and mixed procedures [3,18]. Nevertheless, toilet teaching procedures need to be highly individualized, particularly with learners with long learning histories. The ability to be independent on the toilet may lead to increased independence and increased opportunities in the arena of accessing adult services. The current case study evaluated the use of a school-based, multi-component intensive toilet training procedure with a young adult male diagnosed with ASD who had never successfully voided in the toilet.

## 2. Materials and Methods

### 2.1. Participant

Clyde was a White 18-year-old adult male who had been formally diagnosed with ASD and displayed FI and FE but was not formally diagnosed with FI or FE. Clyde was diagnosed with ASD before the diagnostic criteria included the levels of ASD. It is estimated that, if he were diagnosed today, he would be given Level 3 ASD, which means he requires a significant level of support to complete most tasks and exhibits significant levels of behaviors that interfere with learning, social, and other situations. Clyde did not have an IQ score on record. According to a record review, while he did not have a General Adaptive Composite (GAC) score from the Adaptive Behavior Assessment System—Third Edition (ABAS-3) [22], it was reported that his performance in the conceptual, social, and practical domains was well below average, indicating below-average levels of adaptive functioning. He was non-vocal and used an augmentative communication device (e.g., GoTalk, Attainment Company, Inc., Verona, WI, USA) to communicate his needs. He was able to respond to verbal and gestural prompts to guide his behavior.

Clyde displayed several frequently challenging behaviors, including aggression, self-injurious behavior, spitting, inappropriate touching, and head banging, for which he wore a helmet for protection. These challenging behaviors were tracked by the school team. His behavioral and educational team conducted a Functional Behavior Assessment (FBA) and a Functional Analysis (FA) to determine the function of these challenging behaviors and to develop treatment plans for their reduction and to increase adaptive and alternative responses. It was found that the functions of the challenging behaviors were both attention (gaining access to adults) and escape (avoiding non-preferred teacher-directed demands and instructions). Based on the assessment, the team recommended a Behavior Intervention Plan (BIP) to target a reduction in challenging behaviors and increase adaptive and alternative responses. When Clyde engaged in escape-motivated challenging behaviors, he was given more choices throughout the day, tiered reinforcement for cooperating, and the ability to request breaks. When he engaged in attention-motivated behaviors, the school staff put up a mat to block his view of other students and classroom staff, so that he would not receive the feedback of attention from others when engaging in challenging behaviors. These challenging behaviors had a history of being especially prevalent when he was in the bathroom. Clyde required verbal, partial, or full physical assistance when in the bathroom and to complete most other tasks and had never been successfully toilet trained.

Clyde was approaching the end of his educational services and a transition to adult services. His lack of ability to use the toilet independently had the potential to significantly impair his ability to secure an eligibility-based adult services placement and might be a risk to his personal safety. His educational team discussed the priority of learning independent toileting skills, and his parent agreed to allow the intervention to occur at school.

Clyde had attended school at the same agency since he was a preschooler. Throughout the years, attempts to teach him how to appropriately use the toilet independently were unsuccessful due to his severe challenging behaviors. Futhermore, his parent reported that she was unable to toilet train him at home due to a lack of time and support. Clyde wore diapers exclusively in the home, school, and community. At school, Clyde used a toileting schedule where staff would take him to the bathroom every hour to prompt him to void on the toilet, but this was unsuccessful. While in the bathroom at school, Clyde often engaged in severe self-injurious behavior, which included dropping to the floor and banging his head against hard surfaces and led to the staff helping to use mats to protect him while he was exposed and in a vulnerable state.

### 2.2. Setting

Clyde attended a specialized high school for children with neurodevelopmental disabilities, which served as the setting for this case study. He attended school on weekdays and was a student in a classroom with five other students, the classroom teacher, two teacher assistants, one classroom aide, and one behavior support specialist. He received specialized instruction and therapies according to his individualized education plan (IEP). When not at school, he lived at home with his parent who assumed all care.

Teaching sessions began in the bathroom, which was closed to the other students who were directed to another bathroom in the building. Padding was installed on most hard surfaces in the bathroom to protect Clyde if he engaged in self-injurious behaviors. His entire work area from his classroom was moved into the bathroom stall and included his desk, chair, all work materials, helmet (to implement his behavior intervention plan), and data sheets for toileting. All the surfaces were sanitized, and meals and snacks were eaten in the stall but away from the toilet. When Clyde was successful in the bathroom, the sessions then moved to the classroom, so that he had to walk to the bathroom to use the toilet.

Before the procedure was implemented, the researcher reviewed the procedure with the school nurse, who indicated that the procedure was acceptable and that the risks to Clyde were minimal. The nurse obtained a prescription from Clyde’s physician to increase his fluid intake throughout the day. Before, Clyde was only drinking fluids during the following times: meals, snack time, or when requested via his device. However, once we started to increase fluids, he started to intake a small plastic cup of juice or water every half hour. After his initial intensive toilet training in the bathroom, we started to increase the half-hour to fluids every hour in the classroom. Clyde’s physician was also in support of the intensive toilet treatment package.

### 2.3. Preference Assessment

A pairwise preference assessment was conducted to establish Clyde’s preference for the edible reinforcement [23]. Based on previous assessments conducted by Clyde’s classroom team, as well as observations of his preferences, the edibles were chosen over tangible items as they were deemed stronger, primary reinforcers. It is important to note that, due to the limitations of his motor skills, tangible items were of limited preference. Clyde’s edible preferences did not change throughout the intervention. The items used in the pairwise edible preference assessment included Fritos, pretzels, Doritos, Oreos, Swedish Fish, and Lays Potato Chips. The researcher showed Clyde an option of two snacks and then documented whether he chose one snack over the other, whether he chose both, or neither. The results of the preference assessment showed that Fritos chips were his most preferred edible. The researcher determined this would be the edible distributed to him during a successful void and he would not have access to these chips at any other time. Clyde’s second preferred edible were pretzels, which were provided to Clyde after a successful dry pants check and at no other time. If Clyde were to request a snack, he could have options such as chips or cookies, instead of pretzels and Fritos.

### 2.4. Response Measurement

Success was defined as sitting on the toilet appropriately and voiding urine and remaining free from accidents. An accident was defined as urinating in places other than in the toilet, including in his pants or a diaper. During the baseline and intervention, the staff documented on a data sheet whether he had success on the toilet or an accident in his underwear or diaper and whether he self-initiated to use the toilet with his GoTalk.

### 2.5. Components of the Intervention

Because Clyde had never been taught to use the toilet independently, the researcher implemented the features of toilet training intervention that had been successful in the literature. This included increased fluid intake, the use of a urine alarm, dry pants checks, and an intensive toilet training package to maximize the potential for him to be successful. Additionally, this package included an intensive toileting schedule, the transition from bathroom-implemented intervention to classroom-implemented intervention, and the transition from diapers to underwear.

The urine alarm (Wet-Stop) was clipped to the inside of his diaper. When Clyde began to urinate, the alarm would sound. When staff heard the alarm, they would immediately prompt Clyde to the toilet, allowing him the opportunity to finish his void in the toilet. As soon as Clyde started to urinate in the toilet, the staff member placed a Frito chip in front of him and provided verbal praise.

Dry checks were completed by staff using hand-over-hand prompts to assist Clyde in checking his own pants near the groin area to determine whether it was dry. If Clyde had a successful dry check, meaning his pants were dry, he would be provided a pretzel and verbal praise. If his pants were wet, he was told in a “matter of fact” tone that he was wet and was changed to clean clothing.

Because this toilet training procedure contained many components and multiple classroom staff were involved, behavior skills training (BST) was used to teach the staff how to implement each step of the training package. BST includes skill-based instruction, modeling, rehearsal, and feedback [24]. During the instruction, the classroom staff were given guidelines that detailed the toilet training procedure. After the instruction, the researcher modeled each component of the procedure and how to collect the data. Next, the classroom staff were observed implementing each component of the intervention with Clyde. After the observations were completed, the researcher provided feedback to the staff, outlining what parts of the procedures they did and did not correctly implement. The modeling and rehearsal steps were repeated until all four staff members completed each component of the toileting procedure with 100% accuracy.

During the BST, the staff were provided with an outline of the components to follow when conducting all steps of the toilet training procedure (see Table 1). The researcher observed each staff member performing all parts of the procedure until all the components were conducted with complete accuracy.

### 2.6. Design and Procedure

A case study, with an ABCD design, was conducted with multiple conditions in the final phase. This design was modified based on the participant’s success and needs during the toilet training process (see Table 2). Every time Clyde went to the bathroom, whether initiated or prompted by the staff, the staff would prompt Clyde to push the toilet button on his communication device. In the ABCD design, Phase A represented the baseline phase where typical “business as usual” procedures were used in the classroom. Phase B was conducted in the classroom and included increased fluids, dry pants checks, reinforcement for successful voids, and the use of a urine alarm. Phase C included “Intensive Toilet Training” at the school where Clyde’s day was spent in the bathroom, fluids being increased, and frequent dry checks continuing. During Phase D, Clyde moved back into the classroom and continued his intensive toileting schedule. Phase D included multiple sub-steps. During phase D1, Clyde wore underwear instead of a diaper under his clothes in the classroom. During phase D2, Clyde wore diapers under his clothes in the classroom due to his parent’s request. Then, Clyde returned to Phase D1 where he wore underwear under his clothes in the classroom. During phases C and D, a systematic fading procedure was implemented as indicated in Table 3. If Clyde had two successes urinating in the toilet, he moved to the next step the following school day. If Clyde had an accident, he moved back to the previous step.

### 2.7. Interobserver Agreement

During the preference assessment conducted, two observers collected data with a 100% agreement on the edible preferences of the participant. During the two days of intensive toilet training conducted all day in the bathroom stall, two observers collected data with 100% agreement on successes and accidents. When the participant moved into the classroom for the rest of the procedure, interobserver agreement was assessed on 50% of sessions with 100% agreement on successes and accidents.

### 2.8. Fidelity and Social Validity

A staff survey (see Appendix A) was provided to all the staff that worked with Clyde during the intervention. The survey included questions such as the following: before the intervention, what was the student’s ability to effectively participate in all aspects of toileting? and what specific aspects has the intervention helped the student with? The purpose of the survey was to analyze how effective the intervention was while determining whether it was socially valid as well. In total, 100% of the staff that filled out the survey reported that the intervention was successful in teaching the participant how to appropriately void in the toilet; the staff also reported that it was beneficial as he is only getting older and it will open many avenues for him in the future.

A parent interview (see Appendix B) was conducted prior to the intervention and post-intervention to determine the before and after impact of the procedure. The interview included questions such as the following: has your child ever independently voided in the toilet? and how many times has your child voided successfully in the toilet in the past month? The purpose of the interview was to determine whether the parent saw any purpose and success with the effects of the intervention implemented. Clyde’s parent reported she was very happy about the progress he made in school but wished she had the same success with him at home. She believed this study was needed because it will benefit him for many years.

## 3. Results

**Figure 1:**
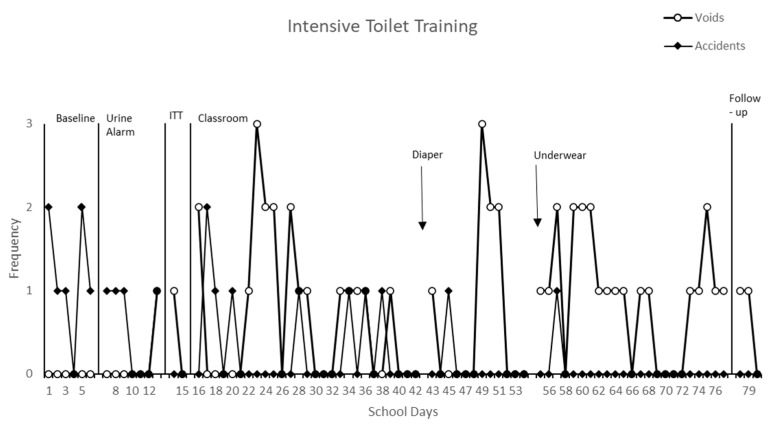
The first solid line indicates the introduction of the urine alarm, the second solid line indicates the introduction of intensive toilet training (ITT) in the bathroom, and the third solid line indicates moving the toileting schedule into the classroom. A follow-up was conducted a month after the intervention was complete.

During the five-day baseline (Phase A), Clyde was having accidents and had no successful voids in the toilet. There was an average of one accident per day with a moderate level and variable trend (see Figure 1). The data also reflect zero successes per day with a low level and stable trend (see Figure 1).

After the baseline, the first intervention phase (Phase B) included increased fluids, including water and his favorite juice; dry pants checks; reinforcement for successful voids; and the use of a urine alarm, as indicated by the first solid phase line in Figure 1. During this phase, Clyde’s average number of accidents was at a moderate level and showed a decreasing trend, and he was successful for three out of seven days. For the three consecutive days, Clyde did not have any accidents. This was a good improvement, but further intervention was required for Clyde to not only not have accidents but to also display successful voiding in the toilet. During Phase B, he did not void on the toilet and his accidents reflect a low level and stable trend.

During the next phase (Phase C), Clyde spent two school days in the bathroom where the intensive toileting schedule was implemented (see Table 3, Step 1), fluid continued to be increased, and frequent dry checks continued. While in the bathroom, Clyde had zero accidents as reflected by the low level and stable trend during Phase C in Figure 1. While the voids show a low level and decreasing trend, he had his first successful void in the toilet.

Phase D began after the third day of Phase C. During Phase D, Clyde moved back into the classroom and continued his intensive toileting schedule. Phase D was broken into sub-steps D-1a, D-2, and D-1b. At first, both accidents and successes were varied and sporadic, but, as time progressed, the number of accidents decreased and the amount of successes increased, remaining consistent. If Clyde had two successes, he moved to the next step in the intensive toileting fading procedure the next day. If Clyde had an accident, he moved back to the previous step.

While Clyde was showing success with the intensive toilet training package, his parent was still very hesitant about the treatment. She claimed that the underwear was providing a rash that would not go away and therefore requested that Clyde go back to diapers while continuing the toilet training. His parent applied an ointment to address the rash. When Clyde went back to diapers at the request of his parent (indicated by an arrow on Figure 1), but was still prompted to follow the toileting schedule of the procedure, he remained successful; Clyde had an accident on one out of twelve days and either no void or successes on eleven out of twelve days. His success continued when Clyde went back to wearing underwear, with his parent’s permission (indicated by the second dotted condition change line, Phase D1 on Figure 1), and his success continued to the end of the procedure. Toward the end of the intervention, Clyde did not have an accident for 21 days. Clyde ranged from zero to two successful voids in the toilet per day and zero accidents per day (see Figure 1). Clyde’s accidents were at a low level and stable trend while the voids were at a moderate level with a variable trend.

Data were collected a month after the study concluded, and it was found that Clyde continued to wear underwear, as well as urinate and have bowel movements in the toilet successfully with no accidents. It was reported that he would get up from what he was doing and walk toward the bathroom in an attempt to self-initiate. After a month, the intervention was still effective. Remarkably, during the entire course of intervention, Clyde displayed only one instance of self-injurious behavior.

## 4. Discussion

Overall, the results indicated that the toilet training procedure was effective for Clyde as he had more successes than accidents and maintained the level of successes throughout the intervention. Clyde’s success was attributed to the rich schedule of reinforcement in place for voiding in the toilet. The results of this case study extend the findings of the existing literature, which primarily includes children as participants, to an adult with limited adaptive skills and significant challenging behavior. Implementing an intensive toilet training package was effective in reducing the number of toileting accidents and increasing the number of successes in the toilet in a young adult with ASD. This study utilized procedures commonly found to be effective in previous studies with children, such as increased hydration, a urine alarm, an intensive bathroom schedule in the bathroom, a bathroom schedule in the classroom, positive reinforcement, and dry pants checks [2]. In addition, behavior skills training, which has been used effectively to teach parents to implement toilet training [25], was successfully used to train classroom staff to implement the procedures. These results are important, because they demonstrate the effectiveness of these strategies, previously shown to be effective with children, to increase an older individual’s independence with toileting, improving his likelihood of being accepted into an adult services program. The ability to independently toilet allows for an increased ability to participate in social situations and a reduced possibility of being alone with staff in a vulnerable situation.

This case study with one participant and no experimental control has limited generalizability but is an important contribution to the literature in that it shows a successful procedure conducted on an adult with ASD who has displayed significant levels of challenging behavior and limited levels of adaptive behavior. Typical methods may not be as effective as specialized behavioral methods of toilet training to address FI and FE in adults with ASD. Further, for a young adult who has gone such a long period without toilet training, with an 18-year history of urinating and having bowel movements in a diaper, typical methods may not work and could lead to frustration, which could be dangerous for an individual who displays extreme challenging behavior. This study may inform researchers and practitioners to help other adults with ASD to successfully learn how to use the toilet, regardless of their age or developmental disability.

Future studies should expand on the current study by implementing the controls of an experimental design and generalizing gains across settings. A component analysis should also be conducted to analyze two or more components within the intervention package. Additionally, future studies should focus on the participant’s communication strategies to establish the initiations to use the toilet. Ideally, a study including multiple young adults with ASD and other IDDs from diverse backgrounds who display FE and FI should be taught to use the toilet to further evaluate the utility of this intervention.

While the toilet training intervention successfully addressed Clyde’s FI, its success can be attributed to the significant support provided to him by the school. However, given the number of staff involved and despite being trained with BST, the staff were not always able to strictly adhere to the toileting schedule. Functionally, there was sometimes between a one- and three-minute delay in prompting Clyde to the toilet. It is equally important to note that, with this limitation, regardless of the delay, Clyde did not have an accident.

Another notable limitation is that, while it was offered, Clyde’s parent did not allow the researcher to implement the procedure at home; therefore, there was no generalization between school and home. Clyde was in school for six hours a day and therefore spent eighteen hours at or on the way to and from home. His parent did not want him to wear underwear when he was at home. Instead, he wore diapers, and it was reported that he continued to have frequent accidents at home.

Despite the listed limitations, this toilet training procedure was effective with Clyde’s ability to urinate in the toilet and reduce his FI. This is remarkable due to the participant’s age and level of maladaptive behaviors. He was consistently successful with the procedure. While traditional toilet training procedures were initiated when the participant was younger, this was the first attempt to implement intensive toilet training to teach him to use the toilet. Since his parent had discontinued the process during his early elementary years, it was pivotal to emphasize the importance of increased independence and the ability to participate in outings. In fact, the participant successfully participated in van rides and field trips while wearing underwear and staying dry. It should also be noted that, once during the intervention, he was on the toilet about to urinate when the fire alarm rang. He got up and went outside, waited for the drill to be over, went back into the school, sat on the toilet, and successfully urinated.

Another notable strength was that, in addition to addressing FI with successful urine voids on the toilet, Clyde started to have successful bowel movements in the toilet. Furthermore, while not measured, the student’s initiation levels increased during the study as he was being taught to self-initiate by requesting the toilet with his GoTalk.

## 5. Conclusions

Increasing independence with toileting is an important life skill. This intervention successfully taught the participant how to use the toilet effectively for urination and bowel movements. This result will improve the participant’s ability to secure an eligibility-based adult services placement, reduce the need to purchase costly diapers, and lead to improved personal safety while needing less help in the bathroom. While the participant was older than the usual age at which children are toilet trained, this procedure was successful for him. It is important that service providers attempt to help participants achieve the greatest level of independence possible, which can lead to more opportunities.

## Figures and Tables

**Table 1:** Components for toilet training procedures.

5 min dry check ^1^
Prompt Clyde to the toilet
Prompt Clyde to press the toilet button on his GoTalk
If Clyde voids, staff provide Fritos with high praise
Go to “off-toilet” activities ^1^

^1^ The duration of time for the dry checks and the duration of “off-toilet” time increased systematically over time and based on Clyde’s success, as described below.

**Table 2:** Phase descriptions and toileting schedule.

Phase Label	Phase Title	Phase Description	Toileting Schedule Step in Phase (See Table 3)
Phase A	Baseline	A data sheet was used for the staff to collect data on the times Clyde was going to the bathroom, whether he had a success or an accident, and whether he initiated the toilet or not.	Toileting schedule not yet used.
Phase B	Urine Alarm	The staff continued to document the student’s toileting habits but a urine alarm was clipped to the inside of his diaper. Whenever Clyde started to urinate, the alarm would sound. As soon as staff heard the alarm, they would prompt Clyde to the bathroom, allowing him the opportunity to finish his void in the toilet.	Toileting schedule not yet used.
Phase C	Intensive Toilet Training	Clyde spent two entire school days in the bathroom following a toileting schedule.	Step 1
Phase D	Classroom with Toileting Schedule	D1a: underwear (Clyde wore underwear at all times while in school).	D1a: steps 1–5.
		D2: diaper (at the request of the parent, he went back to wearing diapers).	D2: steps 5–8.
		D1b: underwear (he began to wear underwear again).	D1b: steps 8–13.

**Table 3:** Toileting schedule ^1^.

Step	Time On/Off Toilet	Dry Checks
1	5 min sitting on the toilet, 5 min off the toilet	5 min
2	5 min sitting on the toilet, 10 min off the toilet	5 min
3	5 min sitting on the toilet, 20 min off the toilet	5 min
4	5 min sitting on the toilet, 30 min off the toilet	5 min
5	5 min sitting on the toilet, 40 min off the toilet	5 min
6	5 min sitting on the toilet, 50 min off the toilet	5 min
7	5 min sitting on the toilet, 60 min off the toilet	5 min
8	5 min sitting on the toilet, 70 min off the toilet	10 min
9	5 min sitting on the toilet, 80 min off the toilet	10 min
10	5 min sitting on the toilet, 90 min off the toilet	15 min
11	5 min sitting on the toilet, 100 min off the toilet	15 min
12	5 min sitting on the toilet, 110 min off the toilet	20 min
13	5 min sitting on the toilet, 120 min off the toilet	20 min

^1^ This systematic fading procedure was adapted from LeBlanc et al., 2005 [17].

## Data Availability

The data presented in this study are available on request from the corresponding author due to privacy considerations.
